# Real-time ultrasound-guided balloon dilatational percutaneous tracheostomy is a safe procedure in critically ill patients: an evaluation study

**DOI:** 10.1186/cc12108

**Published:** 2013-03-19

**Authors:** A Taha, A Shafie, M Mostafa, P Wallen, H Hon, R Marktanner

**Affiliations:** 1 Sheikh Khalifa Medical City, Abu Dhabi, United Arab Emirates

## Introduction

Balloon dilatational percutaneous tracheostomy with radial outward dilation minimizes bleeding and injury to tracheal rings [1]. Using the bronchoscope during the puncture of the trachea holds a risk of inaccurate placement, of hypoventilation and of needle puncturing the bronchoscope. This study was conducted in order to evaluate the safety features and feasibility of combining real-time ultrasound-guided puncture of the trachea with visualization of the needle path during tracheal puncture [2]. We provide precise localization of tracheal cartilage and proper site puncture for the stoma by ultrasound, with subsequent performance of balloon dilatational percutaneous tracheostomy using the Blue dolphin technique.

## Methods

Twenty-five patients including 15 males and 10 females, mean age 61 years, with age range from 23 to 102 years, underwent bedside percutaneous tracheostomy combining real-time ultrasound and the Blue Dolphin technique.

## Results

The median time for the procedure was 15 (12 to 20) minutes. Targeted placement for the tracheostoma between the second and third or the third and fourth tracheal ring was achieved in 100%. No significant complications (for example, tracheal bleeding, puncture posterior tracheal wall, misplacement of the tracheal cannula) occurred. One fractured tracheal ring was identified using bronchoscopy after the procedure. No conversion into a bronchoscopically guided or into a surgical open technique was necessary.

## Conclusion

This study demonstrates the feasibility and safeness combining real-time ultrasound guidance and balloon dilatational percutaneous tracheostomy (Blue Dolphin technique) in critically ill patients. In our division, this technique has become the standard bedside tracheostomy procedure because it combines excellent patient safety features with avoidance of intraprocedural tube misplacement, hypoventilation and accidental bronchoscopy damage even in technically difficult cases [3].
